# RNA Biological Characteristics at the Peak of Cell Death in Different Hereditary Retinal Degeneration Mutants

**DOI:** 10.3389/fgene.2021.728791

**Published:** 2021-10-29

**Authors:** Chunling Wei, Yan Li, Xiaoxiao Feng, Zhulin Hu, François Paquet-Durand, Kangwei Jiao

**Affiliations:** ^1^ Kunming Medical University, Kunming, China; ^2^ Department of Ophthalmology, Affiliated Hospital of Yunnan University, Yunnan University, Kunming, China; ^3^ Key Laboratory of Yunnan Province, Yunnan Eye Institute, Kunming, China; ^4^ Institute for Ophthalmic Research, Eberhard-Karls-Universität Tübingen, Tübingen, Germany

**Keywords:** inherited retinal degeneration (IRD), RNA-seq, biogenic analysis, cGMP-related genes, photoreceptor cell death

## Abstract

**Purpose:** The present work investigated changes in the gene expression, molecular mechanisms, and pathogenesis of inherited retinal degeneration (RD) in three different disease models, to identify predictive biomarkers for their varied phenotypes and to provide a better scientific basis for their diagnosis, treatment, and prevention.

**Methods:** Differentially expressed genes (DEGs) between retinal tissue from RD mouse models obtained during the photoreceptor cell death peak period (*Pde6b* ^*rd1*^ at post-natal (PN) day 13, *Pde6b* ^*rd10*^ at PN23, *Prph* ^*rd2*^ at PN29) and retinal tissue from C3H wild-type mice were identified using Illumina high-throughput RNA-sequencing. Co-expression gene modules were identified using a combination of GO and KEGG enrichment analyses and gene co-expression network analysis. CircRNA-miRNA-mRNA network interactions were studied by genome-wide circRNA screening.

**Results:** *Pde6b* ^*rd1*^ *, Pde6b* ^*rd10*^ *, and Prph* ^*rd2*^ mice had 1,926, 3,096, and 375 DEGs, respectively. Genes related to ion channels, stress, inflammatory processes, tumor necrosis factor (TNF) production, and microglial cell activation were up-regulated, while genes related to endoplasmic reticulum regulation, metabolism, and homeostasis were down-regulated. Differential expression of transcription factors and non-coding RNAs generally implicated in other human diseases was detected (e.g., glaucoma, diabetic retinopathy, and inherited retinal degeneration). CircRNA-miRNA-mRNA network analysis indicated that these factors may be involved in photoreceptor cell death. Moreover, excessive cGMP accumulation causes photoreceptor cell death, and cGMP-related genes were generally affected by different pathogenic gene mutations.

**Conclusion:** We screened genes and pathways related to photoreceptor cell death. Additionally, up-stream regulatory factors, such as transcription factors and non-coding RNA and their interaction networks were analyzed. Furthermore, RNAs involved in RD were functionally annotated. Overall, this study lays a foundation for future studies on photoreceptor cell death mechanisms.

## Introduction

Retinitis pigmentosa (RP) is a set of heterogeneous inherited retinal degenerations (RDs), with a prevalence of approximately 1/4,000 globally (Zhang, 2016). The disease can be caused by mutations in over 65 genes and may show a variety of inheritance patterns, including x-linked inheritance (Salvetti et al., 2021), autosomal dominant congenital (Sarkar et al., 2020), autosomal recessive (Owczarek-Lipska et al., 2020), and mitochondrial (Kim et al., 2018). In a few cases digenic inheritance has also been reported (Gao et al., 2017). Most mutated genes in RP are expressed in rod photoreceptor cells, leading to rod cell death, night blindness, and loss of peripheral vision in early disease stages. Secondary death of cone photoreceptor cells (Veleri et al., 2015), with complete loss of vision, occurs in late-stage RP. At present, treatment developments have included gene (Ducloyer et al., 2020) and cell (Akiba et al., 2020) therapies, as well as subretinal prosthetic device implantation (Chow, 2013). However, each of these therapeutic approaches has its limitations, and no general RP treatment exists at present. Therefore, studying photoreceptor cell death mechanisms to devise novel methods to prevent retinal degeneration progress may aid effective RP and RD treatment. Of special interest might be changes in transcriptional activity that may precede the execution of cell death.

While the genetic causes of RD have often been established, the cellular mechanisms of photoreceptor cell death triggered by those genetic defects are still poorly understood. In many cases exceedingly high intracellular cGMP levels were observed in RD photoreceptors (Wang et al., 2017; Power et al., 2020) and continuous cGMP elevation likely results in over-activation of cyclic nucleotide gated cation channels (CNGC) and protein kinase G (PKG). CNGC activation mediates Ca^2+^ influx, which in turn may lead to activation of calpain-type proteases (Arango-Gonzalez et al., 2014), ultimately leading to photoreceptor cell death. Associated with high cGMP and PKG activation are the enzymatic activities of histone deacetylases (HDAC) and poly (ADP-Ribose) polymerase (PARP) activation (Sancho-Pelluz and Paquet-Durand, 2012). The latter produces poly (ADP-Ribose) (PAR) polymers which can induce the transfer of mitochondrial apoptosis inducing factor (AIF) to the nucleus, eventually leading to cell death (Sahaboglu et al., 2010). Moreover, PAR polymer production can induce cell death through NAD^**+**^ and ATP depletion (Ying et al., 2005).

Investigating transcriptomic changes at the peak of photoreceptor degeneration has a great significance for fully understanding RD. Here, we used RNA-Seq and bioinformatics to examine the transcriptomic differences between three RD mouse models (*Pde6b* ^*rd1*^, *Pde6b* ^*rd10*^, and *Prph* ^*rd2*^), at their respective peaks of photoreceptor cell death, to obtain insights into the pathogenesis of RD caused by different gene mutations, with special attention to genes related to cGMP, PARP, and their upstream factors. Differential expression of transcription factors and non-coding RNAs may be involved in photoreceptor cell death. Moreover, as excessive cGMP accumulation causes photoreceptor cell death, cGMP-related genes were generally affected by different pathogenic gene mutations.

## Methods

### Animal Procedures

RD mouse models (C3H *Pde6b* ^*rd1*^, C3H *Prph* ^*rd2*^, and C57Bl6/J *Pde6b* ^*rd10*^) as well as C3H mice carrying the wild-type (wt) alleles for *Pde6b* and *Prph2* were used irrespective of gender. All four animal colonies were regularly genotyped to ensure that they indeed carried (or not) the disease-causing mutations. The use of congenic C3H mice (Sanyal and Bal, 1973) facilitates comparative transcriptomic analysis between wild-type, *Pde6b* ^*rd1*^ and *Prph* ^*rd2*^. While the *Pde6b* ^rd10^ animals were not congenic to the C3H wt, the impact of the disease-causing mutation is so dramatic that relatively minor strain differences are unlikely to cause a large impact on gene expression patterns. All procedures were performed in compliance with the ARVO statement for the use of animals in Ophthalmic and Visual Research. Protocols were reviewed and approved by the Yunnan University ethical review board. All efforts were made to minimize the suffering and number of animals used. Retinal tissue was collected during the peak period of photoreceptor cell death (post-natal (PN) day 13 for *Pde6b* ^*rd1*^, PN23 for *Pde6b* ^*rd10*^, PN29 for *Prph* ^*rd2*^), wild-type retinal tissues were collected at corresponding time points.

### Tissue Preparation

All mice (PN5-PN180) were sedated with CO_2_ before decapitation. The eyes were removed after cleaning the heads with 70% ethanol. Subsequently, the eyes marked in the nasal part were incubated in 4% PFA for 45 min at room temperature (RT). This was followed by three washes in PBS (0.1 M; pH 7.4) (10 min each). Then, the eye was cut open along the limbus, and the cornea, lens, and vitreous were removed. The eyecups were then, at RT, treated with serially increasing concentrations of sucrose solution (10 min in 10% sucrose, 20 min in 20% sucrose, 30 min in 30% sucrose) for cryo-protection. Subsequently, the tissues were embedded in O.C.T TM compound (Fisher Healthcare™ Tissue-Plus™ O.C.T. Compound, Waltham, MA, United States) and immediately frozen in liquid nitrogen and stored at −20°C until sectioning. The thickness of sections were 10 um, and the sections were baked at 40°C for 45 min before stored at −20°C or further staining.

### TUNEL (In Situ Cell Death Detection) Assay

Fixed retinal sections dried at 37°C for 40 min were washed in PBS (RT, 15 min). For nuclease inactivation and inhibition of nucleic acid reduction, the slices were incubated in TRIS with Proteinase K (10 μg/ml in 10 mM TRIS-HCL, pH 7.4–8.0; 37°C, 5 min) and then washed thrice with Tris (5 min each). Subsequently, the slices were placed in ethanol-acetic-acid (3:1) mix at −20°C for 5 min and then blocked with blocking solution (1% BSA, 10% NGS, 1% fish gelatin, 0.003% PBST) for 1 h, RT. Afterwards, the sections were stained with TUNEL kit (Fluorescein or TMR; Roche Diagnostics GmbH, Mannheim, Germany) at 37°C according to the manufacturer’s instructions and mounted in Vectashield containing DAPI (Vector, Burlingame, CA, United states).

### RNA Extraction, Library Preparation, and Sequencing

The sequencing was carried out by Shanghai Life Gene Biotechnology Co., Ltd., (Shanghai, China). Total RNA was extracted from the entire retinal tissues of different groups using TRIzol reagent (Invitrogen, South San Francisco, CA, United States) following the manufacturer’s instructions. After measuring the quantity and quality of total RNA, a total amount of 3 µg RNA per sample was used for library preparation. Briefly, ribosomal RNA was removed, and sequencing libraries were generated using the rRNA-depleted RNA by NEBNext^®^ Ultra™ Directional RNA Library Prep Kit for Illumina^®^ (NEB, United States) following manufacturer’s recommendations. After library preparation, the total RNA of different groups was then sequenced on the Illumina HiSeq 4,000 platform. The raw data and GEO accession number for this study are as follows: (GSE178928, https://www.ncbi.nlm.nih.gov/geo/query/acc.cgi?acc=GSE178928).

### Quality Control, Alignment, and Quantification of RNA-Seq Data

Clean data were obtained from in-house Perl scripts by removing reads containing adapter, reads containing poly-N, and low-quality reads from FASTQ raw data. At the same time, Q20, Q30, and GC content of the clean data were calculated to evaluate the quality of sequencing. Subsequently, we aligned the clean reads to the mouse reference genome GRCm38. After that, HTSeq v0.11.2 was used to count the read numbers mapped to each gene, and then Fragments Per Kilobase of transcript sequence per Millions (FPKM) of each gene was calculated.

### Identification of Differentially Expressed Genes

DEGs between two groups (C3H vs. *Pde6b* ^*rd1*^, C3H vs. *Prph* ^*rd2*^ and C3H vs. *C57Bl6/J Pde6b* ^*rd10*^) were identified using DESeq2 R package (1.28.0). *p*-value < 0.05 and fold change ≥ 1.5 were set as the thresholds for DEGs. Then, these identified DEGs were submitted for Gene Ontology (GO) and Kyoto Encyclopedia of Genes and Genomes (KEGG) pathway enrichment analyses. A *p*-value < 0.05 was recognized as indicating significant enrichment.

### Functional Enrichment Analysis

Gene Ontology (GO) and Kyoto Encyclopedia of Genes and Genomes (KEGG) pathway enrichment analyses for DEGs were performed using the DAVID (v6.8) and KOBAS (v3.0) software, respectively. *p* value < 0.05 was recognized as indicating significant enrichment.

### CircRNA Annotation and Identification of Differentially Expressed CircRNAs

The obtained clean reads were aligned to the mouse genome (mm10) using TopHat2 (v2.1.1). The CIRCexplorer program (v2.2.3) was used with the fusion junctions obtained from TopHat2 to identify both the circularizing junction and the spliced sequence of circRNAs. All candidate circRNAs with junction reads less than two were discarded. CircRNA expression level was determined by transcripts per million (TPM). DE-circRNAs between two groups (C3H vs. *Pde6b* ^*rd1*^, C3H vs. *Prph* ^*rd2*^ and C3H vs. C57Bl6/J *Pde6b* ^*rd10*^) were screened using the limma R package (3.40.6), and changes were considered as significant with *p*-value < 0.05 and fold change ≥ 2.

### CeRNA Network Construction

The candidate miRNAs were predicted based on miRBase (www.mirbase.org). Targetscan (v7.1) was used to predict the downstream target genes of these miRNAs. The circRNA-miRNA interactions and cicrRNA-mRNA interactions were selected for ceRNA construction using the Cytoscape v3.7.1 software. The genes in the ceRNA networks were then submitted for GO and KEGG enrichment analyses.

### Weighted Correlation Network Analysis

WGCNA (Langfelder and Horvath, 2008) was used for scale-free network topology analysis. Standard WGCNA parameters were used for analysis, with the exceptions of soft-thresholding power and the deep split. Using WGCNA, a co-expression module was quickly extracted for subsequent analysis. In brief, genes with expression correlation were clustered into a module. First, the correlation matrix between two genes was constructed using the main connecting rod and the Pearson correlation matrix. Second, hierarchical clustering analysis was performed using the hclust R function, and the soft thresholding power was determined by analysis of network topology. The adjacency was transformed into a topological overlap matrix. Subsequently, network construction and module detection were performed. Modules with a size less than 30 were merged into one module. The calculation process was performed using the blockwiseConsensusModules function of the WGCNA package.

### Real-Time Quantitative PCR

We chose seven differentially expressed genes obtained by sequencing for further validation. The total RNA was reversely transcribed into cDNA using PrimeScript™ II 1st Strand cDNA synthesis Kit (Takara Biomedical Technology Co., Ltd.). The sequences of all primers are shown in Supplementary Table S4. The total volume of RT-qPCR was 20 μL, including 10 µL SYBR Premix EX Taq, 1 µL forward primer (10 µM), 1 µL reverse primer (10 µM), 2 µL cDNA and 6 µL RNase free water. The RT-qPCR reaction were initiated at 95°C for 10 min, followed by a total of 40 cycles of 95°C for 15 s and 60°C for 30 s. GAPDH served as a housekeeping gene, and the levels of *Xist*, *Foxg1*, H2K2, *Maff*, *Klf6*, mmu-cicr0000135, and mmu-cicr0008206 were calculated using the 2^−ΔΔCt^ method.

### Immunofluorescence Assay

The cryosections were washed in PBS for 15 mins, and then used for antigen retrieval, after that, sections were incubated with anti-PITX2 antibody, (1:200, GB112180, Servicebio, Wuhan, China) anti AIF (1:200, GB11314, Servicebio, Wuhan, China), at 4°C overnight. On the next day, the sections were incubated with the secondary antibody (FITC-labeled 488 goat anti rabbit IgG, 1:300 Servicebio) in the dark at RT for 50 mins. After washing with PBS, the sections were stained with DAPI (Servicebio) in the dark for 10 mins, and then spontaneous fluorescence quencher (Servicebio) was added for 5 mins. After rinsing under running water for 10 min, the sections were sealed with an anti-fluorescence quencher (Servicebio), and then were observed under a fluorescence microscope and confocal Microscope (LSM 900 Airyscan 2, Zeiss, Oberkochen, Germany).

### Microscopy, Cell Counting, and Statistical Analysis

Light and fluorescence microscopy was performed with a Zeiss Imager M2 Microscope and LSM 900 Airyscan two equipped with a Zeiss Axiocam digital camera (Zeiss, Oberkochen, Germany). Images were captured using the Zeiss Axiovision 4.7 software, and representative pictures were obtained from central areas of the retina. Adobe Illustrator CC 2019 (Adobe Systems Incorporated, San Jose, CA) was used for primary image processing. For cell quantification, whole radial slice pictures were captured using the Mosaix mode of Axiovision 4.7. Labelled cells were counted manually. The total number of cells was determined by dividing the outer nuclear layer (ONL) area by the average cell size. The total number of ONL cells was determined by dividing the percentage of positive cells by the number of positive cells. Three sections each from at least three different animals were tested for each genotype and experimental condition. Statistical comparisons between experimental groups were made using one-way ANOVA and Bonferroni’s correction using Prism eight for Mac OS (Graph Pad Software, La Jolla, CA). Values are presented as mean ± standard deviation (SD) or standard error of means (SEM). Levels of significance were as follows: n.s. > 0.05; *, *p* < 0.05; **, *p* < 0.01; and ***, *p* < 0.001.

## Results

### The Peak of Photoreceptor Cell Death in Different RD Mutants *in vivo*

Apoptosis relates to the orderly process of changes in cell morphology and biochemistry that finally results in cell death (Kerr and Thompson, 1972). Aside from apoptosis a variety of programmed cell death mechanisms are known, including programmed necrosis, necroptosis, and PARthanatos (Leist and Jaattela, 2001). TUNEL (Terminal deoxynucleotidyl transferees dUTP nick end labeling) is a common method to detect DNA fragmentation as end result of apoptosis but also of other forms of cell death, for instance in necrosis (Grasl-Kraupp et al., 1995; Loo, 2002). We used the TUNEL cell death assay to identify photoreceptor cell death in these three genetically distinct RD models to assess to what extent universal gene expression changes might underlie photoreceptor degeneration.

The first animal model was the *Pde6b* ^*rd1*^ mouse, one of the most studied animal models for early onset and rapid rod photoreceptor degeneration (Keeler, 1924). The *Pde6b* ^*rd1*^ mouse carries a retroviral insertion in the rod *Pde6b* gene that leads to non-sense mediated mRNA decay and hence no functional PDE6B protein is made (Pittler and Baehr, 1991). As the second animal model, we employed the *Prph* ^*rd2*^ mouse, also referred to as *rds* or “retinal degeneration slow”, which harbors a mutation in the *Prph2* gene that leads to an absence of photoreceptor outer segments (Goldberg, 2006). The third model used was the *Pde6b* ^*rd10*^ mouse (Chang et al., 2007). As opposed to the *rd1* mutation, the *rd10* mutation is a point mutation that affects the catalytic site of the PDE6B enzyme. While this does not abolish protein expression, it dramatically reduces PDE6 activity.

**FIGURE 1:**
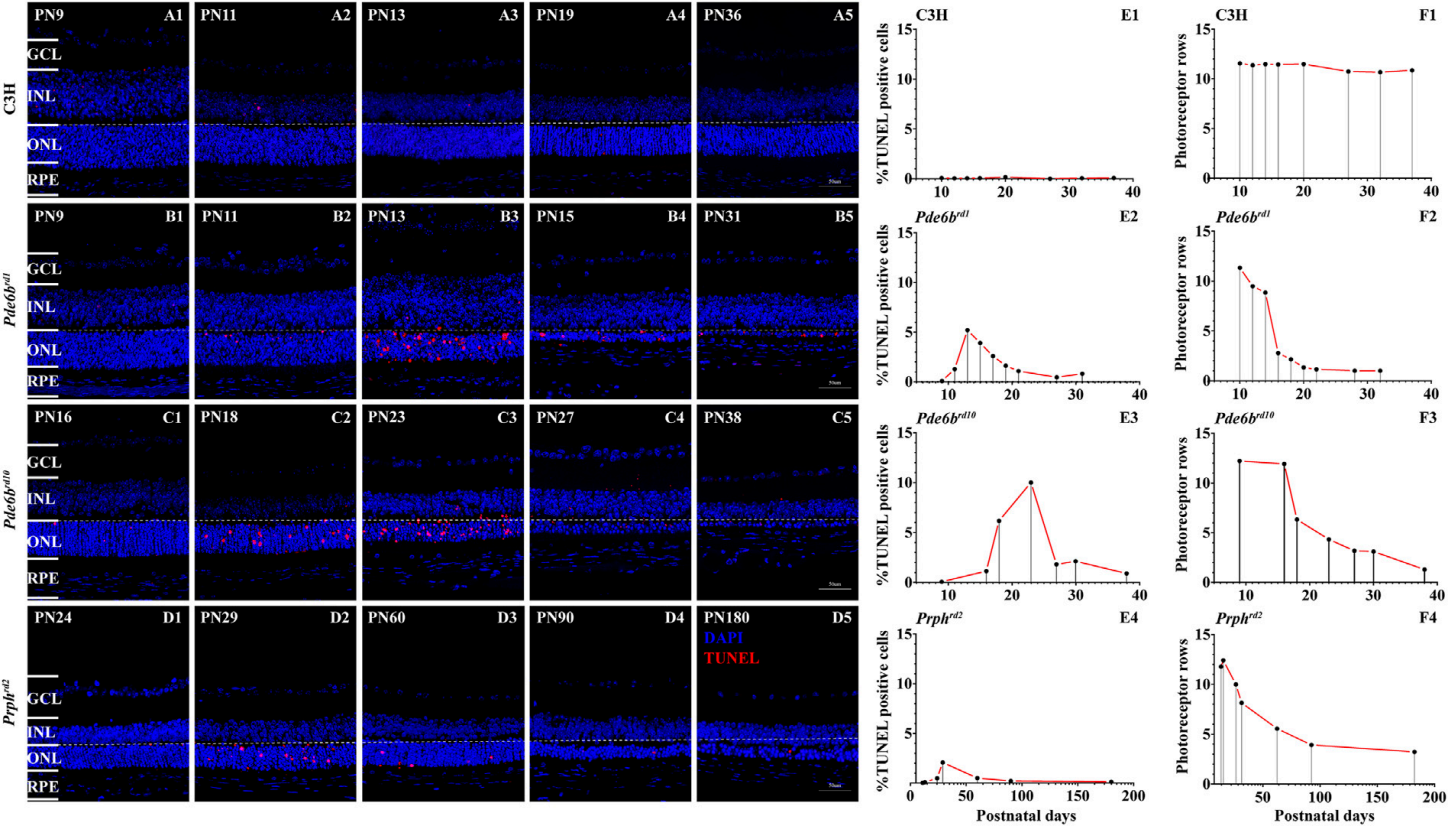
TUNEL assay and cell death in RD mutants. The number of TUNEL positive cells in the three different RD mutants ONL was strongly increased when compared with wt **(A1–D5)**. Quantification of photoreceptor cell death and photoreceptor rows during the first 40 postnatal days in C3H wild-type, *Pde6b* ^*rd1*^, *Pde6b* ^*rd10*^ mutants and 180 days in *Prph* ^*rd2*^ **(A–F)**. Increased numbers of TUNEL positive cells, showing a peak at PN13 in *Pde6b* ^*rd1*^, PN23 in *Pde6b* ^*rd10*^, PN29 in *Prph* ^*rd2*^ was observed **(B1–D5)**. Quantification of photoreceptor rows during the first 40 postnatal days in *Pde6b* ^*rd1*^, *Pde6b* ^*rd10*^, C3H and 180 days in *Prph* ^*rd2*^. In *Pde6b* ^*rd1*^, photoreceptor rows showed the highest number at PN9 (13 ONL cell rows remaining), declining thereafter. In the other two mutants the photoreceptor rows showed the highest number at PN9 in *Pde6b* ^*rd10*^ (12 ONL cell rows remaining) and PN13 in *Prph* ^*rd2*^, declining thereafter. In the RD models, dying cells and photoreceptor cells rows were detectable as late at PN40 in *Pde6b* ^*rd1*^ and *Pde6b* ^*rd10*^ **(B5, C5)**, at PN180 in *Prph* ^*rd2*^ **(D5)**. The values shown originate from three RD mutants from at least three different specimens. Scale bar represents 50 µm.

Our own TUNEL analysis largely confirmed the previous data from the literature. In the *Pde6b* ^*rd1*^ mouse, we found that cell death started at post-natal 9 (PN9), while it began at PN16 in *Pde6b* ^*rd10*^, and at PN11 in *Prph* ^*rd2*^. In all of these RD models, individual dying cells and photoreceptor cells rows were detectable as late as PN31 in *Pde6b* ^*rd1*^, PN38 in *Pde6b* ^*rd10*^ (Figures 1B5,C5), and PN180 in *Prph* ^*rd2*^ (Figure 1D5).

In wild-type (wt) controls, TUNEL positive cells were seen only occasionally in the ONL (Figures 1A1–A5). Compared to wt retinae, *Pde6b* ^*rd1*^ retinae showed a significant elevation of photoreceptor cell death at PN13 (Figures 1B1–B5), and the photoreceptor degeneration was the earliest compared to other two RD mutants. The TUNEL positive cells peaked at PN23 in *Pde6b* ^*rd10*^ while in *Prph* ^*rd2*^ the TUNEL peak was at PN29 (Figures 1C1–C5,D1–D5). All three RD mutants showed significantly higher numbers of TUNEL positive cells than wt (Figures 1E1–E4).

Quantification of photoreceptor rows in *Pde6b* ^*rd1*^ showed the highest number at PN9 (13 ONL cell rows remaining), declining thereafter, with only one ONL cell row remaining at PN21 (Figure 1F2). A slower cell loss was observed for *Pde6b* ^*rd10*^ retinae, which declined from 12 ONL cell rows at PN9 to one ONL cell row at PN38 (Figure 1F3). The *Prph* ^*rd2*^ mutant had 13 photoreceptor rows at PN13, declining to four rows at PN180 (Figure 1F4). In the wt animals photoreceptor row counts remained nearly constant at 11–13 from P9–P38 (Figure 1F1).

### Transcriptomic Characterization of the Three RD Mouse Models

Through sequencing, a total of 522, 228, 400 raw reads were obtained from the 12 samples (triplicates in each group) using the Illumina HiSeq 4,000 platform. After trimming the low-quality paired-end sequences and adapter sequences, we obtained a total of 496, 619, 835 clean reads. At more than 95%, the % of clean reads was very high, with over 10G clean bases in each sample; the single base sequencing error rate was <0.01%. For all twelve sequencing libraries, the lowest Q20 and Q30 were 97.20 and 92.54%, respectively. No GC bias was found (Supplementary Table S1). Subsequently, we aligned the clean reads to the mouse reference genome GRCm38. We found that, in each sample, more than 93% of reads could be aligned to the reference genome, with 87.84–91.02% unique mapping. The clean read percentage and the mapping percentage suggested good sequence quality (Supplementary Table S2). Therefore, all libraries proved to be suitable for further study.

**FIGURE 2:**
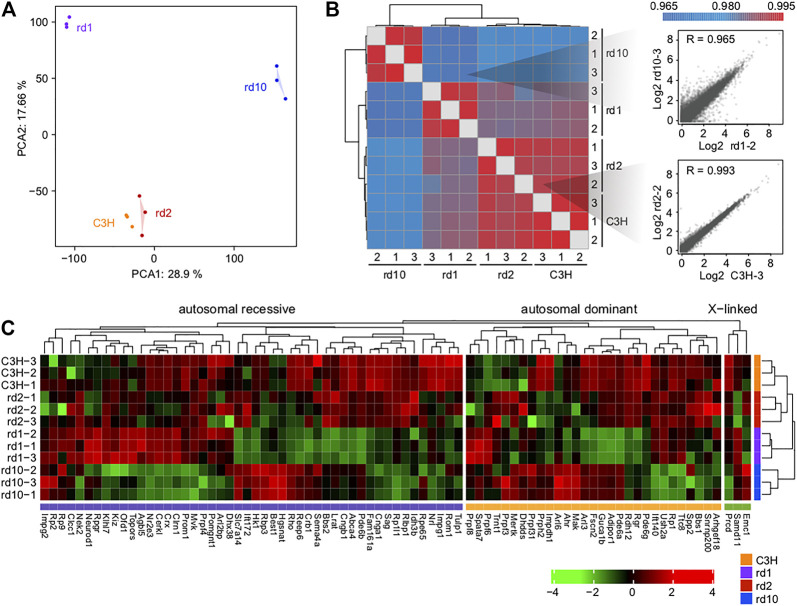
Overall transcriptomic profiles of the three mice models. **(A)** Principal component analysis and **(B)** Pearson correlation coefficients showing the similarity and reproducibility among different biological replicates; **(C)** The expression levels of inherited retinal disease-associated genes in control and RD mice. Note that *rd10* retina expresses *Pde6b* mRNA, albeit for a catalytically inactive protein.

Principal component analysis (PCA) of gene expression profiles showed significant cell type differences (Figure 2A). Pearson correlation analysis revealed that the correlations within each group were stronger than the correlations between groups (Figure 2B). Notably, the *wt* and *Prph* ^*rd2*^ groups had a high correlation and largest degree of similarity, indicating that the gene expression patterns between two groups were more similar. This may correlate with the rather slow progression of *Prph* ^*rd2*^ degeneration which results in relatively few gene expression changes compared to wt.

We searched for genes implicated in RD through RetNet (https://sph.uth.edu/RETNET/) and investigated whether their expression levels were affected in RD mice (Supplementary Table S3). Although disease gene mutations are the leading cause of functional defects in photoreceptors, gene expression imbalance further contributes to photoreceptor degradation. Interestingly, especially in *Pde6b* ^*rd1*^ and *Pde6b* ^*rd10*^ mice, quite a few genes showed a decrease in expression (Figure 2C). This phenomenon suggested that single gene mutations (as in *Pde6b* in the present study) may affect the function of several important genes in various ways.

### Analysis of Photoreceptor Cell Death-Related Genes and Pathways

**FIGURE 3:**
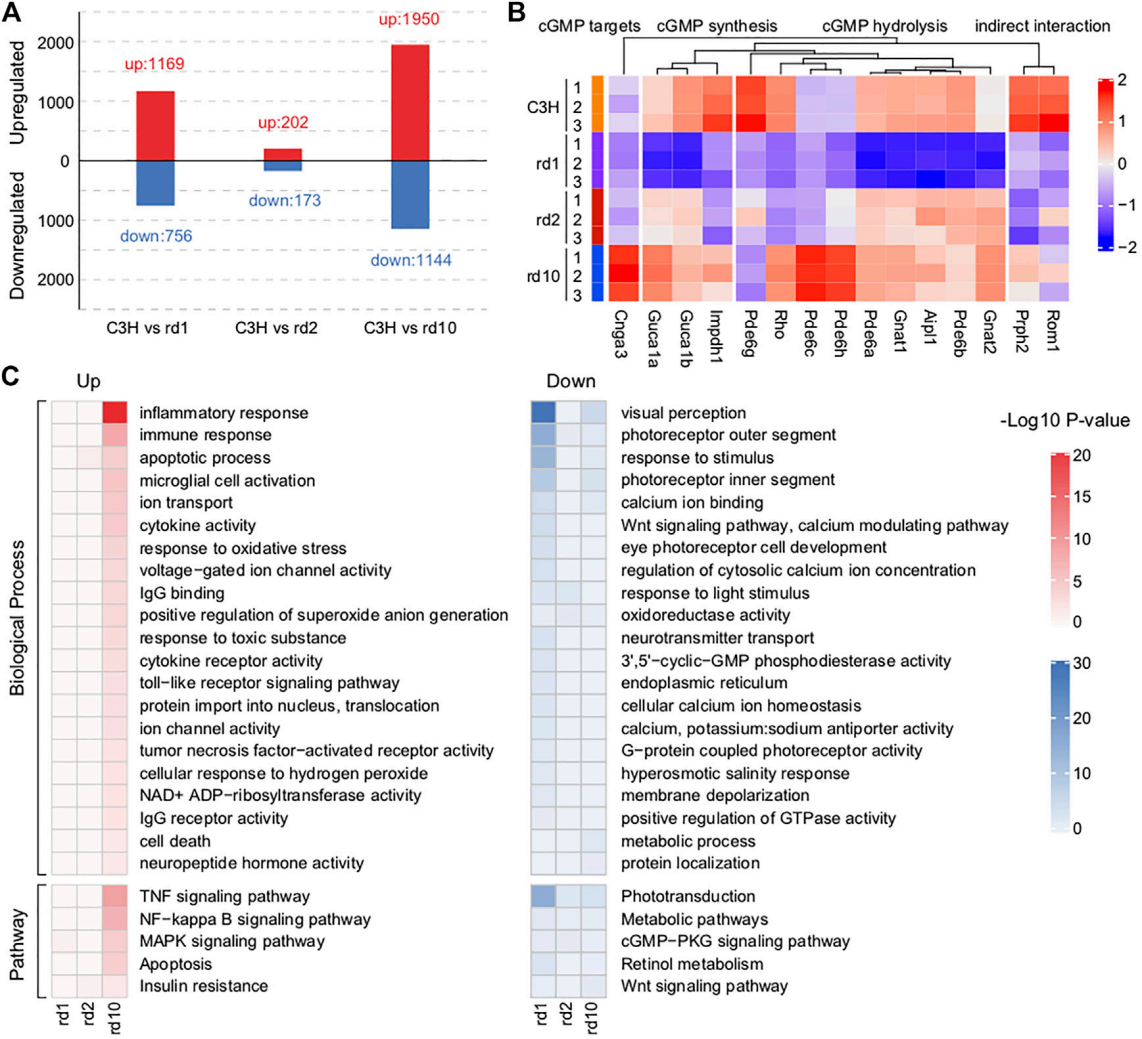
Impaired functions related to phototransduction found in RD mice retina. **(A)** The numbers of differentially expressed genes (DEGs) between C3H mice and each RD model. A total of 1925 DEGs (1,169 up-regulated and 756 down-regulated), 375 DEGs (202 up-regulated and 173 down-regulated), and 3,094 DEGs (1950 up-regulated and 1,144 down-regulated) were respectively identified between *Pde6b* ^*rd1*^ and C3H mice, between *Prph* ^*rd2*^ and C3H mice, and between *Pde6b* ^*rd10*^ and C3H mice. **(B)** Changes in the expression of key genes related to cGMP synthesis, hydrolysis, and targets in RD mice versus that in C3H mice. Genes involved in cGMP synthesis and hydrolysis were generally differentially expressed, especially in the PDE6 mutant *Pde6b* ^*rd1*^ and *Pde6b* ^*rd10*^ mice. **(C)** Functional enrichment analysis of the identified DEGs based on *p* < 0.05. These up-regulated DEGs mainly were enriched ion channel activity, inflammatory process, immune response, TNF signaling pathway, MAPK signaling pathway, and microglial cell activation. The down-regulated DEGs were related to visual perception, Wnt signaling pathway, calcium modulating pathway, positive regulation of GTPase activity, cGMP-PKG signaling pathway and retinal metabolism.

To examine the potential mechanism underlying photoreceptor cell degeneration, we analyzed the DEGs between C3H and RD mice (Figure 3A; Supplementary Figure S1A). Totally, 1,926 DEGs (1,169 upregulated, 756 downregulated) existed between *Pde6b* ^*rd1*^ and C3H. Despite being derived from the same mutant gene Pde6b, *Pde6b* ^*rd10*^ showed 3,096 (1,950 upregulated, 1,144 downregulated) DEGs. However, between *Prph* ^*rd2*^ and wt only 375 (202 upregulated, 173 downregulated) DEGs were identified, indicating the presence of different molecular regulatory mechanisms in the *Prph* ^*rd2*^ model. Unsupervised hierarchical clustering of all DEGs showed clear boundaries among groups with clustering of genes with similar expression behaviors (Supplementary Figures S1B,C).

Among these DEGs, many were known critical regulators of RD. Typically, PDE6 loss-of-function mutations lead to extremely high cGMP levels and death of the affected photoreceptor cell type (Power et al., 2020). Thus, we examined the photoreceptor cGMP-related RD gene expression. Genes involved in cGMP synthesis and hydrolysis were generally differentially expressed, especially in the PDE6 mutant *Pde6b* ^*rd1*^ and *Pde6b* ^*rd10*^ mice (Figure 3B). Obviously, insufficient expression of various PDE6 subunits (*Pde6a*, *Pde6b*, and *Pde6g*) reflected a strong link between gene mutations and transcriptional defects.

Since many dysregulated genes were detected in RD mice, we combined GO and KEGG analyses to further understand the functional defects caused by gene mutations (Figure3C). Highly consistent with the mouse RD phenotype, the activation of apoptosis-related functions and the degeneration of the photoreceptors were observed. However, only a few DEGs were identified in *Prph* ^*rd2*^ mice (Figures 3A,C). Therefore, we focused on general functional associations determined through the study of *Pde6b* ^*rd1*^ and *Pde6b* ^*rd10*^ mice. In summary, the GO terms mainly enriched among upregulated genes were ion channels, stress, inflammatory processes, TNF production, and microglia. The GO terms mainly enriched among downregulated genes were related to the regulation of endoplasmic reticulum, metabolism, and intracellular homeostasis. As one of its roles, in the presence of a high cGMP concentration, PDE6 may activate Ca^2+^ ion channels to trigger Ca^2+^ ion influx; the increased intracellular Ca^2+^ may be a major factor leading to photoreceptor degeneration (Dean et al., 2002). In addition to genetic defects, research indicates that oxidative stress, neuroinflammation, and intracellular toxicity may contribute to retinal degeneration progression (Martinez-Fernandez de la Camara et al., 2013; Yoshida et al., 2013). Insufficient energy production and intracellular environment disorders are both hallmark events of cell death (Hassa et al., 2006). Thus, these dysregulated functions are key to explaining RD pathogenesis. Notably, while the above-mentioned functions were more activated in *Pde6b* ^*rd10*^, they were extensively inhibited in *Pde6b* ^*rd1*^ mice, suggesting that mutations at different locations in a single gene could affect the phenotype in different ways. Another interpretation would be that genes regulated in opposing ways between *Pde6b* ^*rd1*^ and *Pde6b* ^*rd10*^ are, in fact, not relevant for the degeneration and were instead due to different post-natal age and developmental stage (i.e., PN13 vs. PN23).

### Roles of Transcription Factors in Regulating RD

**FIGURE 4:**
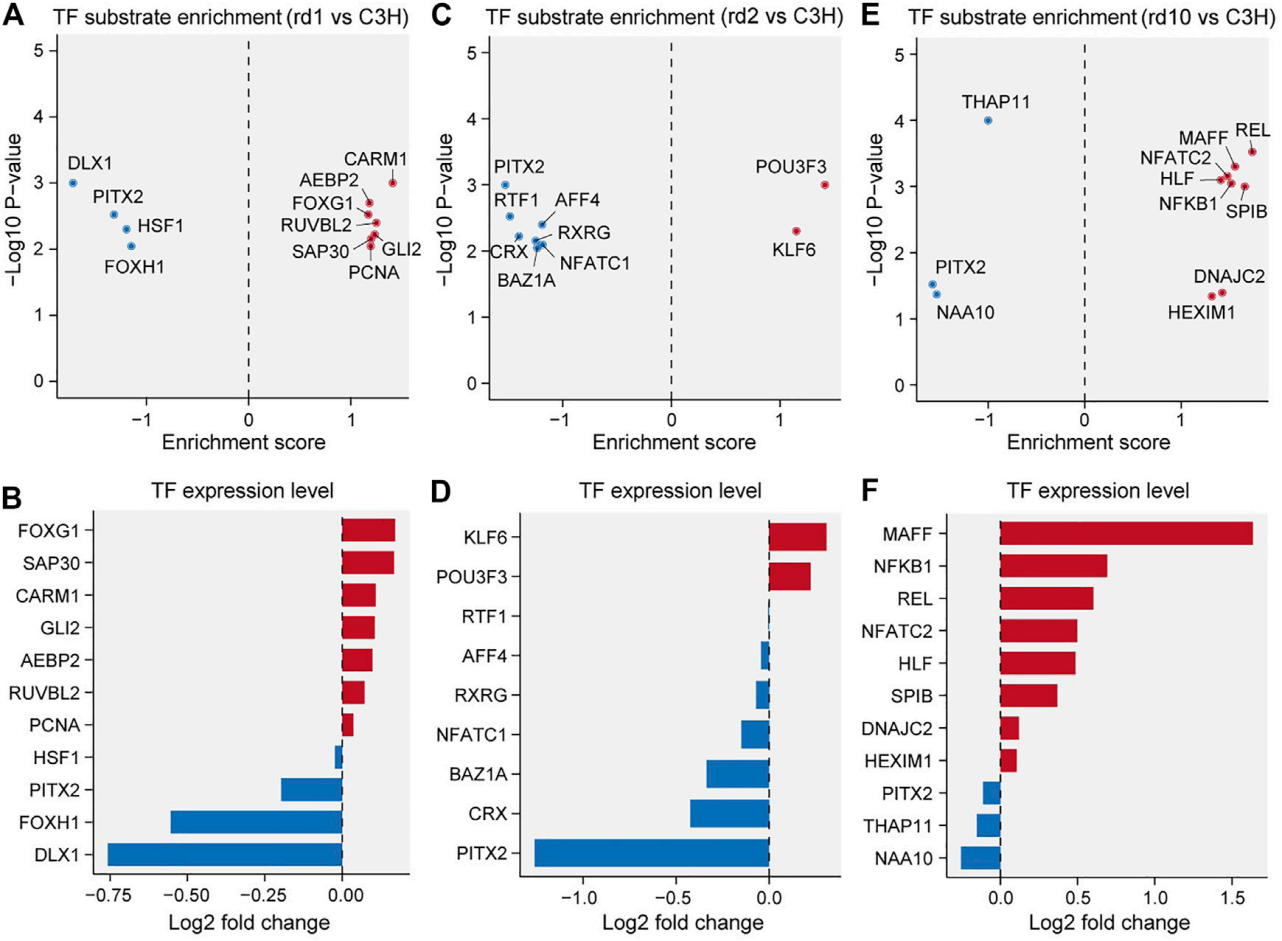
Transcriptional factor (TF) substrate enrichment analysis in the three RD models versus C3H mice**. (A, C, E)** TF substrate enrichment score and significance in *Pde6b* ^*rd1*^ **(B)**, *Prph* ^*rd2*^ **(D)**, and *Pde6b* ^*rd10*^ **(F)** mice; PITX2 were all down-regulated in RD mice compared with the C3H mice. **(B, D, F)** Log2 fold changes of the indicated TF expression levels in *Pde6b* ^*rd1*^ **(B)**, *Prph* ^*rd2*^ **(D)**, and *Pde6b* ^*rd10*^ **(F)** mice. FOXG1, KLF6, and MAFF were up-regulated in RD mice; while DLX1 and NAA10 were down-regulated in RD mice.

To investigate the transcription factor (TF) mechanisms responsible for retina formation and visual nerve development, we extracted genes with significantly altered transcriptional relationships in the three RD mouse models (Figures 4A,C,E). In *Pde6b* ^*rd1*^ and *Pde6b* ^*rd10*^ mice, more TFs were activated, whereas a large area with high TF suppression existed in *Prph* ^*rd2*^ mice. TF enrichment by mutated genes was highly consistent with the TF expression pattern, confirming that the continuous activation or inhibition of upstream signals caused a series of downstream functional changes (Figures 4B,D,F). Notably, *Pitx2* was downregulated in all three RD models, showing the lowest fold change in *Prph* ^*rd2*^ mice, which could be due to its later developmental age at PN29. *Pitx2* in particular has been related to neural crest cell (NCC) survival and migration, as well as optic stalk development, and is critical for eye morphogenesis (Evans and Gage, 2005; Chawla et al., 2016; Hendee et al., 2018). Considering that the functional defect caused by *Pitx2* mutation often induces ocular cell death (Semina et al., 1996), we speculated that the inhibition of *Pitx2* transcriptional activity was also related to the increased risk of neurodegenerative diseases. Compared with *Pitx2*, *Dlx1*, and *Foxg1* had a more significant reduction in expression in *Pde6b* ^*rd1*^ mice. *Dlx1* participates in neurotransmitter transmission and is essential for neuron proliferation, differentiation, and migration; and may regulate cell cycle progression through Wnt signaling (Maira et al., 2010; Wright et al., 2020). *Foxg1* is important for embryonic primitive streak development and reprogramming; its “transcriptional switch” function has received widespread attention in embryonic development (Reid et al., 2016; Wang et al., 2019). The lack of TFs for genes encoding these important proteins would impede optic nerve development. In *Pde6b* ^*rd10*^ mice, *Maff* was highly upregulated. The MAFF protein can regulate photoreceptor gene expression and is closely associated with inflammatory responses in neuronal diseases (Katsuoka et al., 2003; Chevillard et al., 2007; Saliba et al., 2019). We speculate that constant MAFF activation may contribute to inflammatory processes in the microenvironment through pro-inflammatory cytokines or chemokines, thereby mediating photoreceptor cell death. Thus, our data suggests that functional abnormalities of key TFs may accelerate RD pathogenesis.

### Functional Analysis of Differentially Expressed LncRNAs

Long non-coding RNAs (lncRNAs) are involved in gene activation and silencing. LncRNAs have emerged as important regulators of photoreceptor development, as well as of ocular physiology, and their dysregulation is associated with numerous eye-related diseases (Wawrzyniak et al., 2018). Therefore, we extracted molecules annotated as lncRNAs among the identified DEGs, described their expression profile characteristics, and explored the role of important lncRNAs in RD.

In total, 482 differentially expressed lncRNAs (DE lncRNAs) were detected in *Pde6b* ^*rd1*^ mice (312 upregulated and 170 downregulated). *Pde6b* ^*rd10*^ mice showed 728 DE lncRNAs (367 upregulated and 361 downregulated). In *Prph* ^*rd2*^ mice, 101 DE lncRNAs were detected (55 upregulated and 46 downregulated) (Supplementary Figures S2A–C). Similar to the number of DEGs (Figure 3A), *Prph* ^*rd2*^ mice had the lowest number of DE lncRNAs. Unsupervised cluster analysis of DE lncRNAs also showed an evident expression pattern among the three groups (Supplementary Figures S2D,E). In particular, we observed that the gene encoding for X inactive-specific transcript (*Xist*) had the highest degree of upregulation among all three RD mouse models. *Xist* is an X-chromosomal non-coding transcript essential for the initiation and spread of X-inactivation. *Xist* abnormal expression is related to malignancy progression (Xing et al., 2018; Yang et al., 2018; Liu et al., 2019). In retinoblastoma, *Xist* expression dysregulation has been reported to affect cell proliferation and apoptosis (Wang et al., 2018). Overexpressed *Xist* protects human retinal pigment epithelial cells against hyperglycemia-related damage by reducing apoptosis and restoring migration (Dong et al., 2020). Therefore, we speculate that *Xist* overexpression in defense against cell injury may cause apoptotic signaling in RD mouse photoreceptor cells.

**FIGURE 5:**
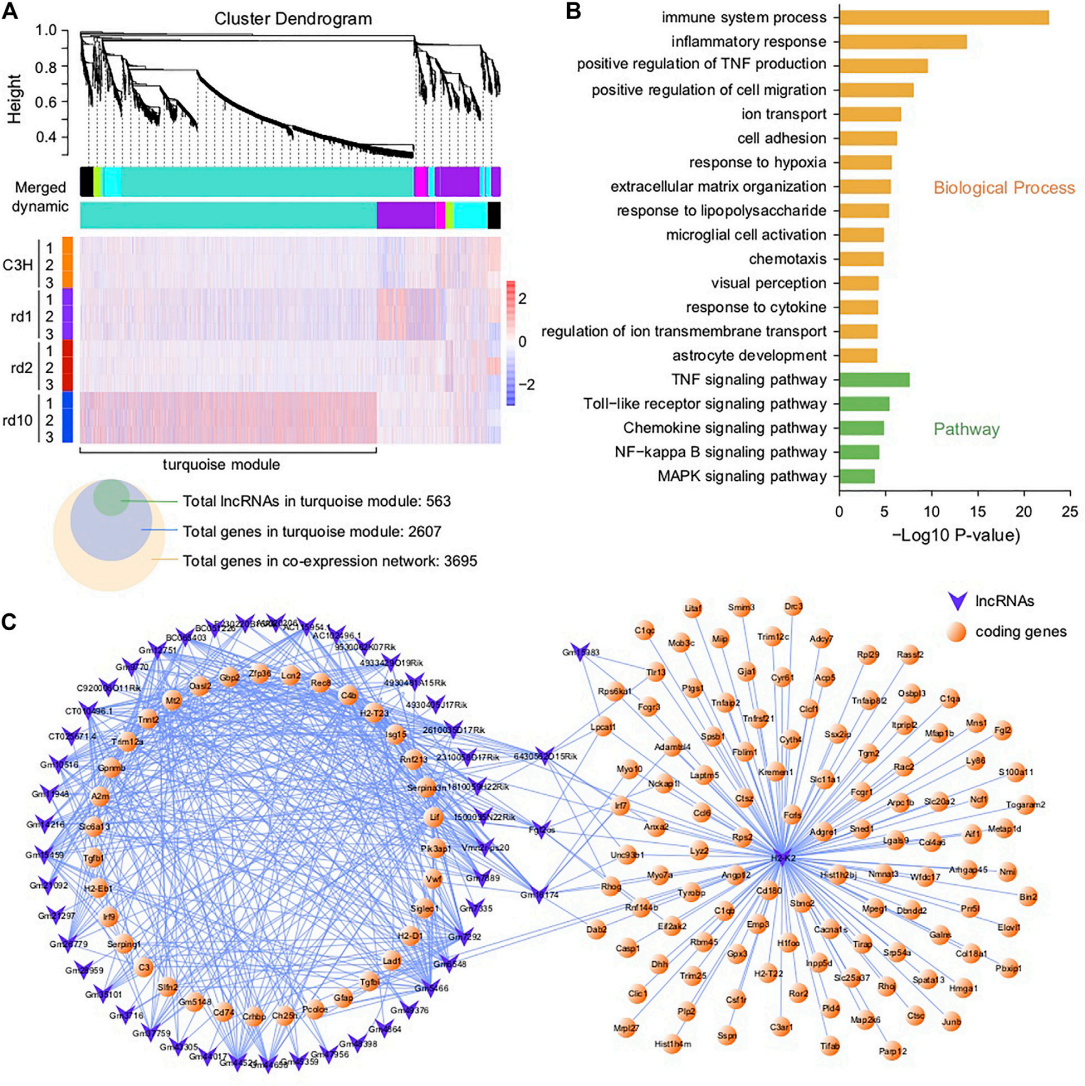
Co-expression network analysis of DE lncRNAs and coding genes. **(A)** Co-expression modules of DEGs and their expression profile in all samples; A total of 563 DE-lncRNAs and 2,607 genes were found in turquoise module, and 3,695 genes in the co-expression network. **(B)** Functional enrichment analysis of DEGs in the turquoise module; The genes in the networks were significantly enriched in immune system process, inflammatory response, positive regulation of TNF production, TNF signaling pathway, Toll-like receptor signaling pathway, chemokine signaling pathway, and MAPK signaling pathway. **(C)** Network connections of lncRNAs with coding genes in the turquoise module—only topological overlap matrices with >0.69 similarity are displayed.

To further understand the regulatory relationship between lncRNAs and coding genes in RD, we performed a weighted gene co-expression network analysis (WGCNA) of all DEGs, including DE lncRNAs. A total of five co-expression modules were observed (Figure 5A, excluding black module). Among these, the module with the largest number of correlated genes (*i.e*., the turquoise module in Figure 5) was the largest and comprised more than 70% of DEGs in the co-expression network, 20% of which were lncRNAs (Figure 5A). This module therefore formed the focus of our subsequent analysis. GO and KEGG analyses showed that functions related to stress response, immunity, inflammation, TNF production, cell migration, chemotaxis, visual perception, and ion transport were extensively enriched in the turquoise module (Figure 5B). Thus, the above biological processes seem to be important in RD, indicating that, in the RD murine gene co-expression network, lncRNAs may generally participate in the regulation of degenerative pathways (Figure 3C). Similarly, several lncRNAs were hub nodes (Figure 5C), likely deserving more attention. For instance, based on the report that lncRNA H2K2 promoted diabetic nephropathy progression via the miR-449a/b/Trim11/Mek signaling pathway (Chen et al., 2019), we inferred that dysregulated H2K2 in RD mice may partially mediate retinal disease progression via the competitive endogenous RNA (ceRNA) mechanism, in which lncRNAs may compete with protein-coding mRNAs for binding to miRNAs. However, the functional roles and regulatory mechanisms of lncRNAs acting as ceRNAs in RD are still unclear.

### Characterization of circRNAs and Construction of ceRNA Network

Previous reports indicate that circRNAs can regulate synaptic activity and are essential for the normal function of the central nervous system (Rybak-Wolf et al., 2015). The retina is a key component of the visual phototransduction system. While circRNAs participate in retinal development, abnormally expressed circRNAs may cause various neurological disorders and diabetic retinopathy (Lukiw, 2013; Shan et al., 2017). However, the circRNA-regulation of retinal degeneration is still unclear. Here, we tried to characterize the circRNA expression profile and the functional relationship of circRNAs with coding DNA in RD mice.

A total of the 12,510 unique circRNAs were identified in our 12 samples (Supplementary Figure S3A). Most circRNAs (7,866, 64.74%) were novel when compared with those in circBase. Notably, the proportion of novel circRNAs in each sample was always less than half (Supplementary Figure S3B), implying that more novel circRNAs have a spatiotemporal pattern in different retinal diseases. Likewise, less than 1/4 circRNAs were shared among the three RD mice models and control mice. Most circRNAs were <1,000 nt long (Supplementary Figure S3C). The distribution of circRNA genomic origins showed that about half of the host genes (1,714, 43.12%) produced only one circRNA, whereas a few host genes (559, 14.06%) produced more than six circRNAs (Supplementary Figure S3D). In general, circRNAs originated from coding exons (Supplementary Figure S3E) and were encoded by less than seven exons (usually two to four) (Supplementary Figure S3F). Additionally, nearly 20% retinal circRNAs in each RD mouse model were conserved in human genome sequences (Supplementary Figure S3G).

**FIGURE 6:**
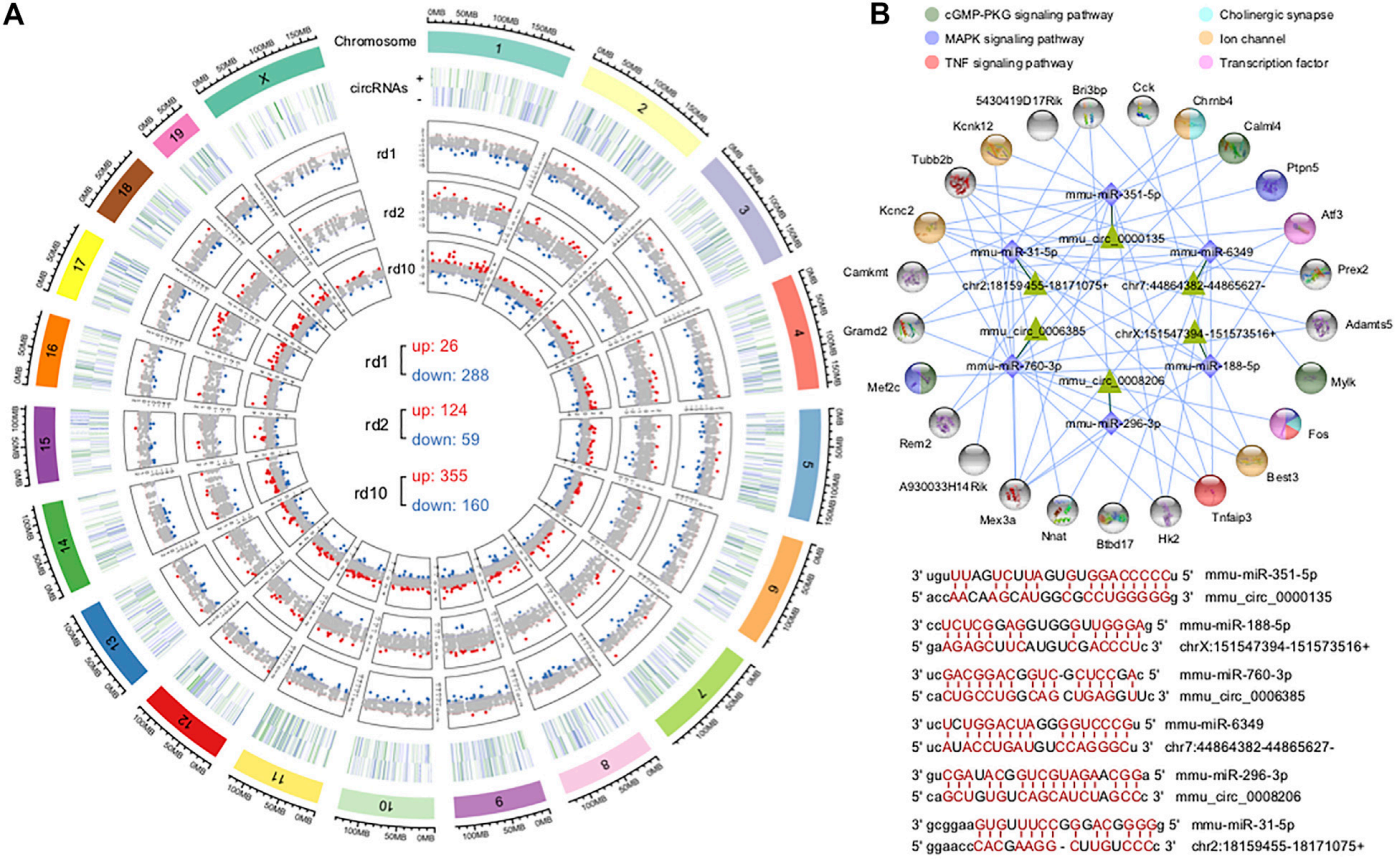
Identification of differentially expressed circRNAs and construction of ceRNA networks. **(A)** Chromosome distribution of circRNAs in mouse retina and their fold changes in the three RD models; There were 314 DE circRNAs (26 up-regulated and 288 down-regulated), 183 DE-circRNAs (124 up-regulated and 59 down-regulated) and 515 DE-cicrRNAs (355 up-regulated and 160 down-regulated) in *Pde6b* ^*rd1*^, *Prph* ^*rd2*^, and *Pde6b* ^*rd10*^. **(B)** The circRNA-miRNA-mRNA interaction network constructed using the common DE circRNAs and DEGs of the three RD mouse models; representative circRNA-miRNA interaction sites predicted by miRanda. These DE circRNAs were enriched in cGMP-PKG signaling pathway, MAPK signaling pathway, TNF signaling pathway, ion channel, transcription factor and cholinergic synapse.

Analysis of differentially expressed circRNAs (DE circRNAs) revealed 314 DE circRNAs in *Pde6b* ^*rd1*^ mice (26 upregulated and 228 downregulated), 515 DE circRNAs in *Pde6b* ^*rd10*^ mice (355 upregulated and 160 circRNAs downregulated), and 183 DE circRNAs in *Prph* ^*rd2*^ mice (124 upregulated and 59 downregulated) (Figure 6A). Interestingly, although the expression levels of most DE circRNAs were specific for different retinal pathological states, almost all the shared DE circRNAs were consistently suppressed (Supplementary Figures S3H,I).

The interaction of circRNAs and miRNAs may indirectly regulate gene expression. Thus, we constructed a ceRNA network using the circRNAs and DEGs of all RD mouse models to explore the circRNA-miRNA-mRNA regulatory axis that may be related to retinal degeneration. The constructed network had 25 genes, 6 circRNAs as decoys, and 6 predicted miRNAs in total (Figure 6B), suggesting that circRNAs can competitively bind to miRNAs through binding sites, thereby indirectly regulating miRNA target genes. Notably, many targets were key genes or genes encoding transcription factors associated with neuronal synapses, ion channels, and inflammation, which may be important in retinopathy pathogenesis. This network provided useful clues indicating that circRNAs play a role in the pathogenesis of retinal degeneration by indirectly targeting certain important genes.

### Validation of Gene Expression Analysis

**FIGURE 7:**
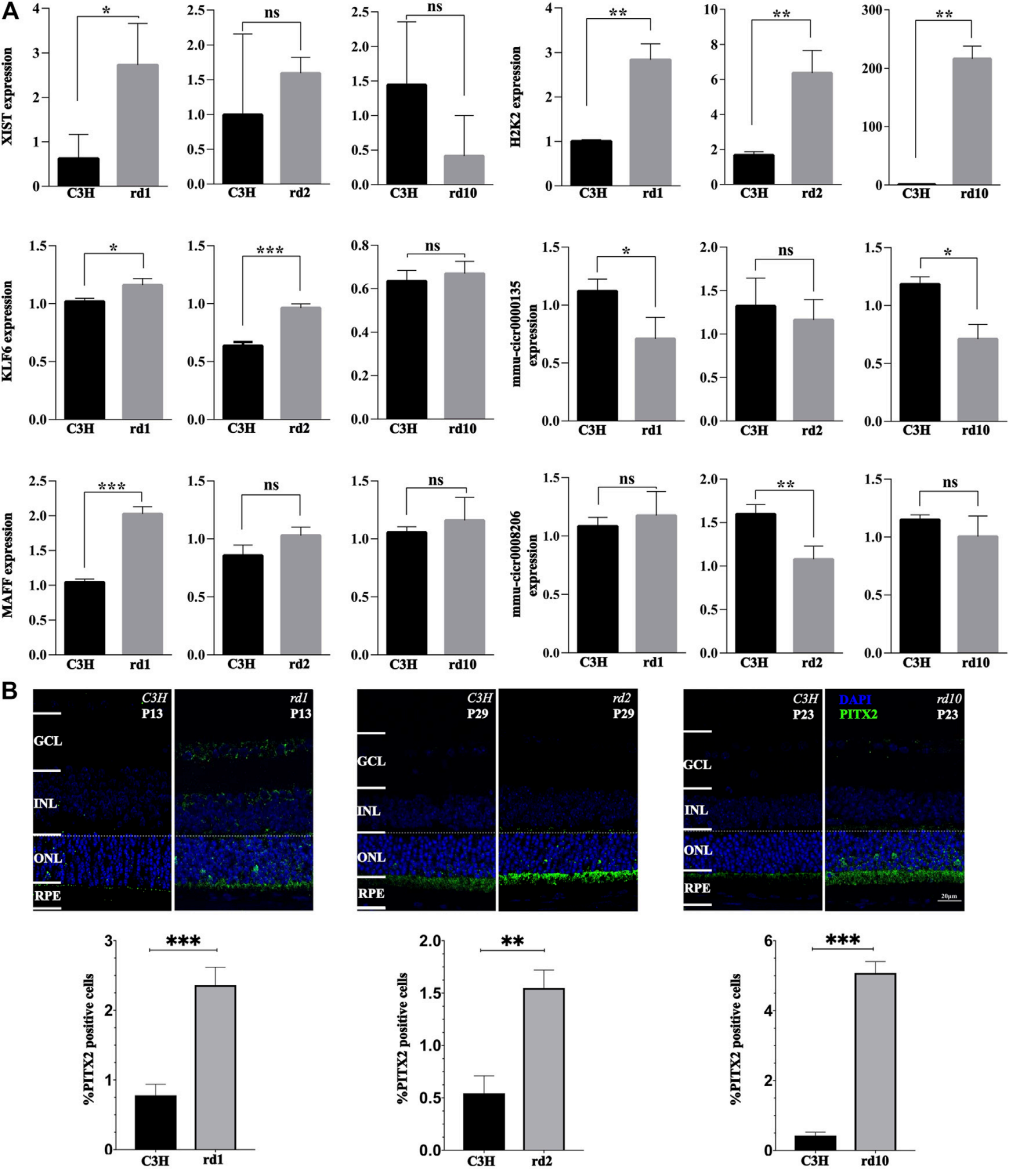
Validation of gene expression changes via qPCR and immunofluorescence. **(A)** The relative levels of XIST, H2K2, MAFF, KLF6, mmu-circ0000135, and mmu-circ0008206 in different groups at different times determined by real-time quantitative PCR. **(B)** PITX2 expression in C3H wild-type, *rd1*, *rd2*, and *rd10* retina at time-points corresponding to the peak of retinal degeneration as assessed by immunofluorescence. Quantification in bar graphs displays the percentage of PITX2 positive cells in the outer nuclear layer (ONL). Significance levels indicated by asterisks: * = *p* < 0.05, ** = *p* < 0.01, *** = *p* < 0.001.

To further validate the gene expression data, we performed RT-qPCR and immunofluorescence staining on retinal sections. While the expression of *Xist* was significantly up-regulated in the *Pde6b* ^*rd1*^ mice at PN13 compared with C3H control (*p* < 0.05), no significant differences were observed in *Xist* expression between C3H control and *Pde6b* ^*rd10*^ mice at PN23, as well as between C3H control and *Pde6b* ^*rd2*^ mice at PN29 (*p* > 0.05, Figure 7A). For H2K2, its expression was evidently up-regulated in all three RD mutant mice when compared to C3H (*p* < 0.05, Figure 7A). *Klf6* was markedly up-regulated in both *Pde6b* ^*rd1*^ and *Pde6b* ^*rd2*^ mice compared with the C3H mice (*p* < 0.05), yet, there was no significant difference in *Klf6* expression between C3H control and *Pde6b* ^*rd10*^ (*p* > 0.05; Figure 7A). Compared with the C3H control, the level of circ0000135 was significantly lower in the *Pde6b* ^*rd1*^ and *Pde6b* ^*rd10*^ mice (*p* < 0.05), while no significant difference was found between C3H and *Pde6b* ^*rd2*^ mice (*p* > 0.05, Figure 7A). The trend of *Maff* gene expression in different groups was similar to that of *Xist* expression, with a significant increase only in the *Pde6b* ^*rd1*^ mouse (Figure 7A). For circ0008206 a significant down-regulation was observed only in *Pde6b* ^*rd2*^ mice (*p* > 0.05, Figure 7A).

In addition, PITX2 protein expression was assessed by immunofluorescence. Remarkably, PITX2 expression was significantly increased in the photoreceptor layer of all three RD mutant mice (*p* < 0.05, Figure 7B). The latter indicated that PITX2 may indeed play an important role in RD pathogenesis.

## Discussion

RNA-Seq, a new-generation sequencing method with a higher accuracy than microarrays, promises a more comprehensive understanding of the changes in gene regulation in RD (Cai and Lyu, 2012). Here, we screened genes and pathways related to photoreceptor cell death in different RD models. Additionally, up-stream regulatory factors, such as transcription factors and non-coding RNA, and their interaction networks were analyzed. Furthermore, RNAs involved in RD were functionally annotated. Overall, this study lays a foundation for future studies on the transcriptional changes leading up to photoreceptor cell death and the discovery of potential targets in the treatment of RD.

### RNA-Seq Transcriptome Profiling of RD Models

During the period of photoreceptor cell death, a previous RNA-Seq study on *Pde6b* ^*rd10*^ mutant retinal tissue showed a lack of gene expression specificity in rod cells and an increased gene transcription in Muller cells and indicated the importance of gliosis in innate immune activation in RD (Uren et al., 2014). In their retinal degeneration study, Xu *et al.* showed that, in *Pde6b* ^*rd1*^ mice, intravitreal metformin injection delayed visual impairment and inhibited photoreceptor cell death (A et al., 2019). Moreover, metformin treatment altered the gene expression profile of *Pde6b* ^*rd1*^ mice. Furthermore, GO and KEGG enrichment analyses showed that immunity and inflammation played a relatively important role in this change (A et al., 2019). Here, since the number of DEGs observed was low and pathway modifications of significance were absent in *Prph* ^*rd2*^ mice (Figures 3A,C), we focused mainly on *Pde6b* ^*rd1*^ and *Pde6b* ^*rd10*^ mice and summarized the PDE6 gene mutations inducing RD pathogenesis.

### Wnt/*β*-Catenin Signaling Protects Photoreceptors From Degradation

Loss of function PDE6 mutation resulted in decreased PDE6*α/β* activity and a high cytoplasmic cGMP concentration (Tsang et al., 1996). Photoreceptor cell physiology relies on the regulation and interplay of the second messenger signaling molecule cGMP and Ca^2+^ levels (Vighi et al., 2018; Das et al., 2021). Classic opsin signaling involves cGMP-gated ion channels; high intracellular cGMP concentrations perpetually open cGMP-gated ion channels, leading to increased Ca^2+^ influx and, ultimately, photoreceptor degeneration (Dean et al., 2002). We found ion channel activity and ion transport to be activated, while cellular Ca^2+^ homeostasis and Ca^2+^-binding mechanisms were impaired, probably leading to excessive intracellular Ca^2+^ accumulation (Figure 3C). Notably, the cGMP-PKG signaling pathway was enriched in downregulated genes.

In addition to causing toxicity and increasing osmotic pressure, high intracellular Ca^2+^ concentrations promote Ca^2+^-dependent calpain-type protease activation, which in turn activates both caspase-dependent and -independent apoptotic pathways (Susin et al., 1999; Mizukoshi et al., 2010). Moreover, mitochondrial calpains can activate AIF, a cell death executioner, which, upon translocation from mitochondria to the nucleus promotes chromatin fragmentation (Polster et al., 2005; Mizukoshi et al., 2010). Curiously, our transcriptomic analysis did not indicate changes in calpain related genes. This may be due to the fact that the rapid metabolic activation by high levels of Ca^2+^ does not affect the transcriptome, most likely because when calpains become activated the cell is already undergoing the final stages of cell death, at which point the transcriptional machinery no longer works. Activated insulin receptors are likely to play a neuroprotective effect by closing cGMP-gated ion channels and reducing Ca^2+^ entry into cells (Rajala et al., 2008; Gupta et al., 2012). Moreover, wnt/*β*-catenin signaling protects photoreceptors from degradation (Patel et al., 2015). Our data showed that insulin resistance pathways were activated, whereas the want signaling pathway was inhibited in RD (Figure 3C). Thus, targeting molecules related to these pathways may rescue photoreceptor cell degeneration.

### X Inactive-Specific Transcript

*Xist* mRNA, as a potential non-coding transcript encoded by the X chromosome and essential for the initiation and spread of X-inactivation, plays an important role in epigenetic processes due to its widespread transcriptional activity. In our RNAseq data *Xist* had the highest degree of up regulation among all three RD models, a finding that was partly confirmed by qPCR. *Xist* abnormal expression is related to malignancy progression (Xing et al., 2018; Yang et al., 2018; Liu et al., 2019). In retinoblastoma, *Xist* expression dysregulation has been reported to affect cell proliferation and apoptosis (Wang et al., 2018). Overexpressed *Xist* protects human retinal pigment epithelial cells against hyperglycemia-related damage by reducing apoptosis and restoring migration (Dong et al., 2020). On the other hand, deletion of the *Xist* gene results in skewed inactivation of the wt X chromosome, indicating that this locus is essential for gene silencing. Our qPCR results showed that *Xist* was up-regulated in the *rd1* and *rd2* mice, indicating that *Xist* may be closely associated with RD pathogenesis. Taken together, we speculate that *Xist* overexpression in defense against cell injury may cause apoptotic signaling in RD mouse photoreceptor cells.

### *Pitx2* Related to RD Pathogenesis

Paired-like homeodomain transcription factor 2 (Pitx2), one of the homeobox transcription factors that play key roles during embryogenesis, has been reported to be crucial in the asymmetric development of the internal organs and the symmetric development of eye tissues (Okubo et al., 2020). *Pitx2* participates in the development of keratocytes, scleral cells, corneal endothelial cells, ciliary muscles, trabecular mesenchymal cells, and peripheral connective tissues connected to the extraocular muscles (Gage et al., 2005; Kimura et al., 2014). Mutations in *PITX2* or *FOXC1* cause Axenfeld-Rieger syndrome, which is characterized by hypoplasia of the anterior segment and mild dental and craniofacial malformations (Seifi et al., 2016). A previous study indicated that SLC13A3 is a direct downstream target of *Pitx2* transcriptional regulation, and the levels of PITX2 and SLC13A3 regulate responses to oxidative stress in ocular cells (Strungaru et al., 2011). Another report showed that deficiency of *Pitx2* could lead to abnormal ocular and craniofacial development in zebrafish (Ji et al., 2016). Our study found that *Pitx2* mRNA was significantly down-regulated in all three RD models. However, immunostaining for PITX2 revealed a strong increase in individual photoreceptor cells in all three RD models. This may indicate an increased translation or stabilization of the *Pitx2* mRNA. Alternatively, the PITX2 protein may be less degraded during the degeneration. In either case the association with the RD models suggests that *Pitx2* play important roles in RD pathogenesis, and further *in vivo* and *in vitro* experiments need to be performed to validate this conclusion.

### Apoptosis-Inducing Factor Activated in Photoreceptor of RD Models

**FIGURE 8:**
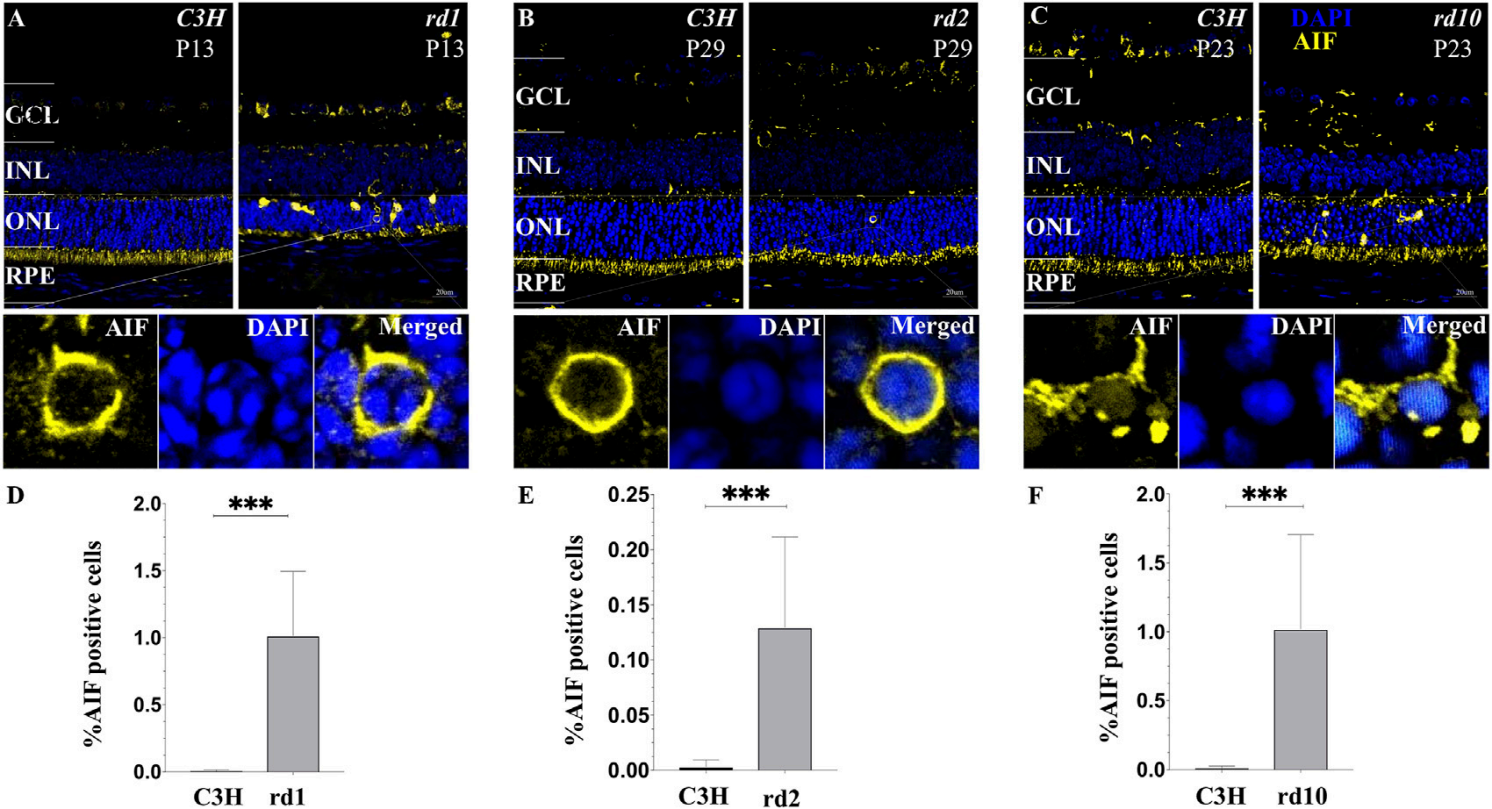
Translocation of AIF in various models of RD photoreceptors. **(A–C)** Confocal microscopy of RD retinal sections stained with AIF (yellow fluorescence) and the nuclear dye DAPI (blue). Cells that demonstrate a clear AIF staining of the nucleus were regarded as positive for AIF translocation. **(D–F)** Note that C3H wild-type control lack any detectable AIF redistribution to the nucleus. The values shown originate from three RD mutants from at least three different specimens. Quantification in bar graphs displays the percentage of AIF positive cells in the outer nuclear layer (ONL). Significance levels indicated by asterisks: * = *p* < 0.05, ** = *p* < 0.01, *** = *p* < 0.001.

Based on our analysis of cell death progression presented above, we chose to study the peak of photoreceptor cell death for all our studies (Figure 1). AIF is a mitochondrial protein usually located in the inner segment of cells containing mitochondria and endoplasmic reticulum (ER) and in the cytoplasm of other retinal cells (Sanges et al., 2006). When stimulated by apoptosis signals, the mature AIF protein translocate into the nucleus, causing DNA breaks and inducing apoptosis. During the hydrolysis of AIF, Calpains (I/II) play a key role in its maturation and release process. When homeostasis of Ca^2+^ is unbalanced in the cells that will cause the number of intracellular Ca^2+^ to rise, calpain activates the hydrolysis and release of AIF (Polster et al., 2005; Badugu et al., 2008). Although, calpain activity may not be reflected at the transcriptional level, we have performed immunofluorescence of AIF to verify if the reallocation of AIF to the nucleus as a result of calpain activity is a confirmed phenomenon. In the stage of peak of photoreceptor cell death, a large nuclear translocation of AIF is found within the RDs photoreceptor segments, areas which are known to host large numbers of mitochondria. However, AIF was not observed in wild type (wt), as determined by confocal analyses (Figures 8A–F), suggesting that AIF plays a primary role in RD cell death event. These data confirm that, before the induction of apoptosis mitochondria stain for AIF, and after the induction of apoptosis, AIF gets redistributed from mitochondria to the nucleus. Because some AIF will be distributed in the cytoplasm, further affecting mitochondrial functions, the increase of mitochondrial membrane permeability leads to an increase in Ca^2+^. So, the translocation of AIF is related to initial cell death changes in the nucleus. The process follows Calpain activation and triggers downstream cell death events (e.g., PARPs) (Plesnila et al., 2004), eventually leading to late cell death as well.

### Identification of PARP Regulated Transcriptomic Signatures

The role of poly (ADP-ribose) polymerase-1 (PARP-1) in responding to DNA damage and genomic stability (Durkacz et al., 1980), maintenance of telomeres (Beneke et al., 2008), and the stability of the replication fork (Bryant et al., 2009) is well known. However, the transcriptional functions of PARP-1 that may relate to chromatin remodeling as a means to regulate DNA repair are unknown (Ikejima et al., 1990). There is evidence that the enzyme and transcription functions of PARP-1 increase as the disease progresses, and are unrelated with DNA repair in cancer (Schiewer et al., 2018). In neurodegenerative diseases, the effects of PARP-mediated photoreceptor degeneration were multifaceted. NAD^+^ is a PARP cofactor. Therefore, sustained PARP activity may reduce NAD^+^ levels, inhibit glycolysis and the TCA cycle, and ultimately decrease ATP production (Goodwin et al., 1978; Bernofsky, 1980), which could lead to an energetic collapse and, subsequently, photoreceptor cell death (Hassa et al., 2006). Moreover, PARP generates PAR polymers, which may mediate AIF nuclear translocation and accelerate DNA defragmentation (Fatokun et al., 2014). Toxicity caused by excessive accumulation of PAR in photoreceptor cells may be another cause of cell death. Consistent with these points, our data indicate increased NAD^+^ ADP-ribosyltransferase activity, nuclear translocation, and cytotoxicity and down regulation of pathways related to metabolism (Figure 3C). Oxidative stress in cells was observed, probably due to the increase in reactive oxygen species (ROS) levels caused by mitochondrial respiration impairment. Oxidative stress plays a pivotal role in retinal degeneration pathogenesis (Lee et al., 2011; Oveson et al., 2011). Interestingly, elevated cGMP levels were found to correlate with increased PARP activity in dying photoreceptor cells (Paquet-Durand et al., 2007; Sahaboglu et al., 2010), implying that the cGMP-mediated cell death process might, to some extent, be related to PARP levels.

Considering the potential impact of PARP mediated function in the process of RD and the need for biomarkers of PARP response, it is particularly important to understand the molecular mechanisms of PARP in different RD models and different ages, and determine the contribution of PARP mediated transcription events to the death of photoreceptor cells. Our study shows that, compared with *wt* mice, the expression of *Parp-1*, *Tank1*, *Parp-6*, *Parp-8*, *Parp-11,* and *Tiparp* was elevated in *Pde6b* ^*rd1*^ retina, while the expression of *Parp-3*, *Parp-4*, *Parp-9*, *Parp-12*, *Parp-14,* and *Parp-16* was elevated in the *Pde6b* ^*rd10*^ situation. However, there was no significant differential expression of PARP genes in *Prph* ^*rd2*^ mice (Supplementary Figure S1D). The latter may be due to the fact that the numbers of dying cells are relatively low in *Prph* ^*rd2*^, even at the peak of cell death. Further investigation of the PARP regulated transcriptome would bring new understanding of PARP and transcriptional functions that are related to the function of RD progression.

PARP cytotoxic levels, stress response, and inflammation are closely linked to a myriad pathological conditions, including in neuroinflammation (Martinez-Fernandez de la Camara et al., 2013; Yoshida et al., 2013). Accordingly, we found that biological processes involved in inflammatory or immune response were dysregulated (Figure 3C). Remarkably, TNF signaling, associated with ocular inflammatory diseases and retinal degeneration (Valentincic et al., 2011; Yoshida et al., 2013), is highly activated in RD mice. TNF is considered both an apoptotic and a necroptotic inducer; it can trigger downstream NF-κB, MAPKs, and apoptotic pathways (Vandenabeele et al., 2010). These pathways were activated in our RD mice (Figure 3C). Inhibition of TNF signaling reduces photoreceptor cell death (Martinez-Fernandez de la Camara et al., 2015). Thus, its inhibition may prove useful in reducing neurodegeneration caused by inflammation and could therefore be an important target in future RD treatments. TNF is secreted in the retina, most likely by activated macrophages or microglia. Genes involved in the positive regulation of leukocyte migration and inflammatory responses were upregulated, which is reflected in increased cellular stress and accelerated TNF release. Furthermore, microglial activation, a neuropathology hallmark and an early event in retinal degeneration and photoreceptor cell death, was observed (Gupta et al., 2003). Therefore, various microglial activation inhibitors and TNF inhibitors have been suggested for RD treatment. For instance, minocycline delayed photoreceptor cell death in an RD mice model (Peng et al., 2014). Furthermore, by specifically blocking the interaction between transmembrane TNF and its receptor, adalimumab protects the neuroretina by reducing microglial cell activation and inhibiting downstream cell apoptosis signaling (Mirshahi et al., 2012; Fernandez-Bueno et al., 2013). Therefore, it can be inferred that PARP regulated transcriptome may play essential roles in RD pathogenesis.

Nevertheless, there are some limitations in this study. Firstly, the specific roles of the identified DEGs in RD pathogenesis need to be further investigated *in vitro* and *in vivo*, and the related mechanisms of PITX2 in RD also should be further explored. Given the potential involvement of *Xist*, experiments it may be of interest to also study the effects of gender in RD.

In conclusion, the photoreceptor cell death mechanism is complex and requires further comprehensive studies. In addition to studying alterations in important genes and pathways involved in RD, we focused on the possible roles of upstream regulators. TFs are essential for initiating gene expression, cell proliferation, differentiation, and cell fate determination. Furthermore, many non-coding RNAs play key roles in the regulation of cell homeostasis (Amaral et al., 2013). In the retina, non-coding RNAs can regulate the expression of genes involved in oxidative stress, ion channels, retinal layer connections, basement membrane integrity, and receptor clusters (Donato et al., 2018), which are all related to RD pathogenesis (Giblin et al., 2016) Accordingly, our results showed that dysregulated TFs or non-coding RNAs, including lncRNAs and circRNAs, may be of importance for photoreceptor cell death and participate in RD pathogenesis by mediating the stress response, apoptotic factor production, ion channel activity, optic nerve signal transduction, metabolism, and homeostasis regulation. Additionally, many TFs and non-coding RNAs (e.g., *Xist*, *Pitx2*, PARPs) were identified as key regulatory molecules associated with RD. In this way, our work lays a foundation to discover and develop novel potential therapeutic targets for treating RD.

## Data Availability

The series entry (GSE178928, https://www.ncbi.nlm.nih.gov/geo/query/acc.cgi?acc=GSE178928) provides access to all of our data and is the accession that can be quoted in any article discussing the data.
