# Supplementary figures and images for: RNA Biological Characteristics at the Peak of Cell Death in Different Hereditary Retinal Degeneration Mutants
Source: Front Genet. 2021 Oct 29;12:728791. doi: 10.3389/fgene.2021.728791 (PMC8586524; doi:10.3389/fgene.2021.728791)

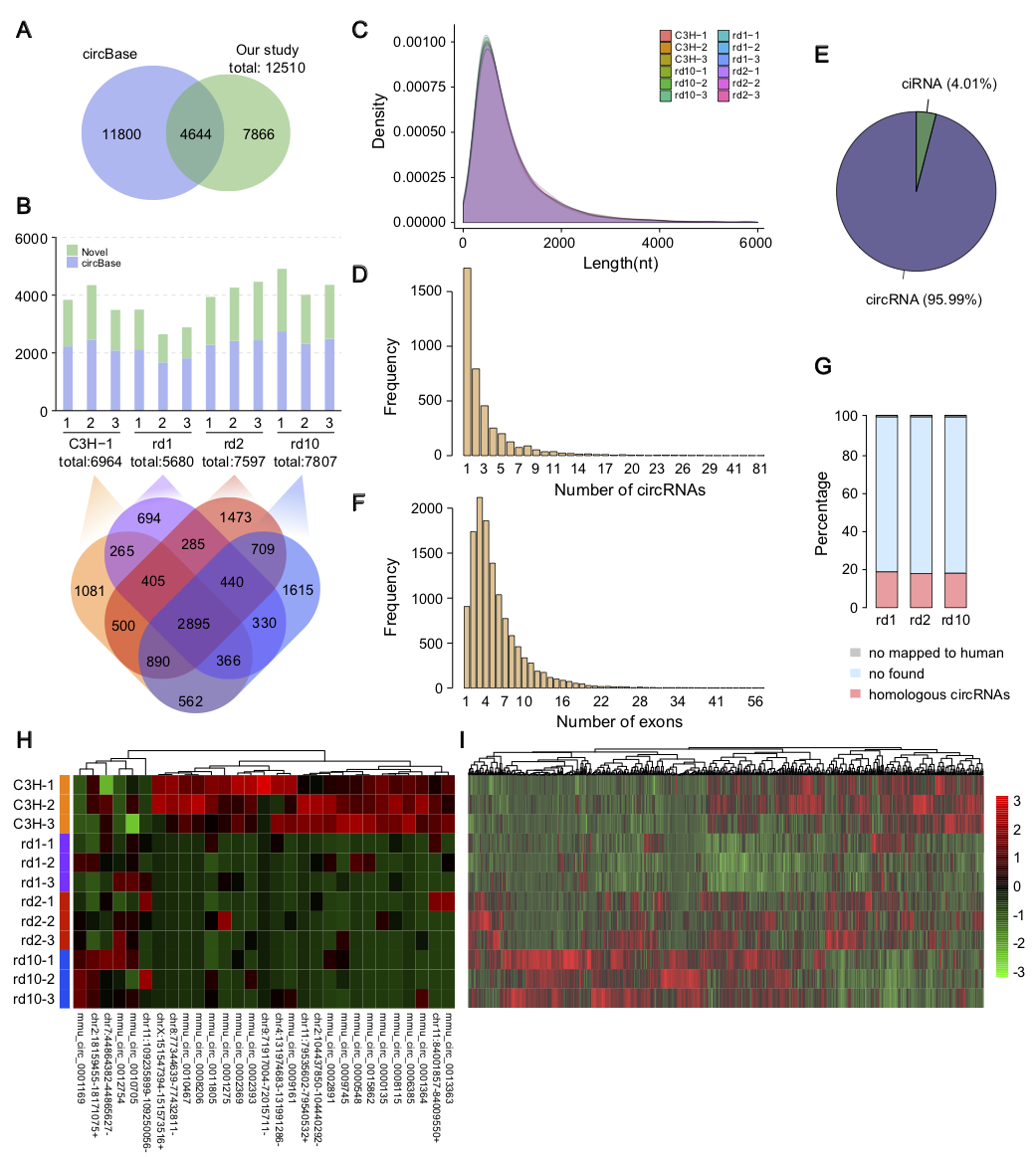

Supplement: Supplementary file 1 [file Image3.JPEG]

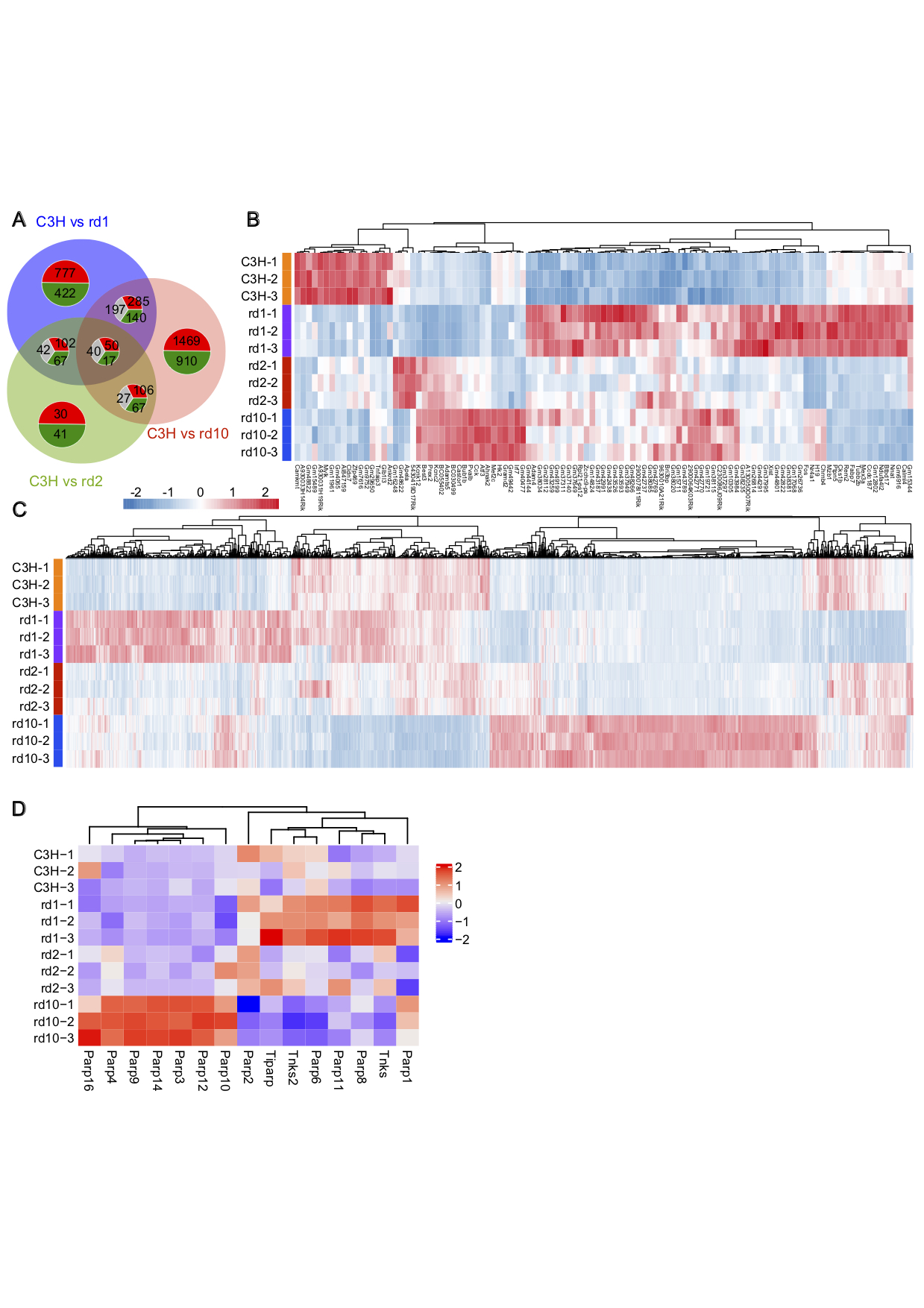

Supplement: Supplementary file 4 [file Image1.JPEG]

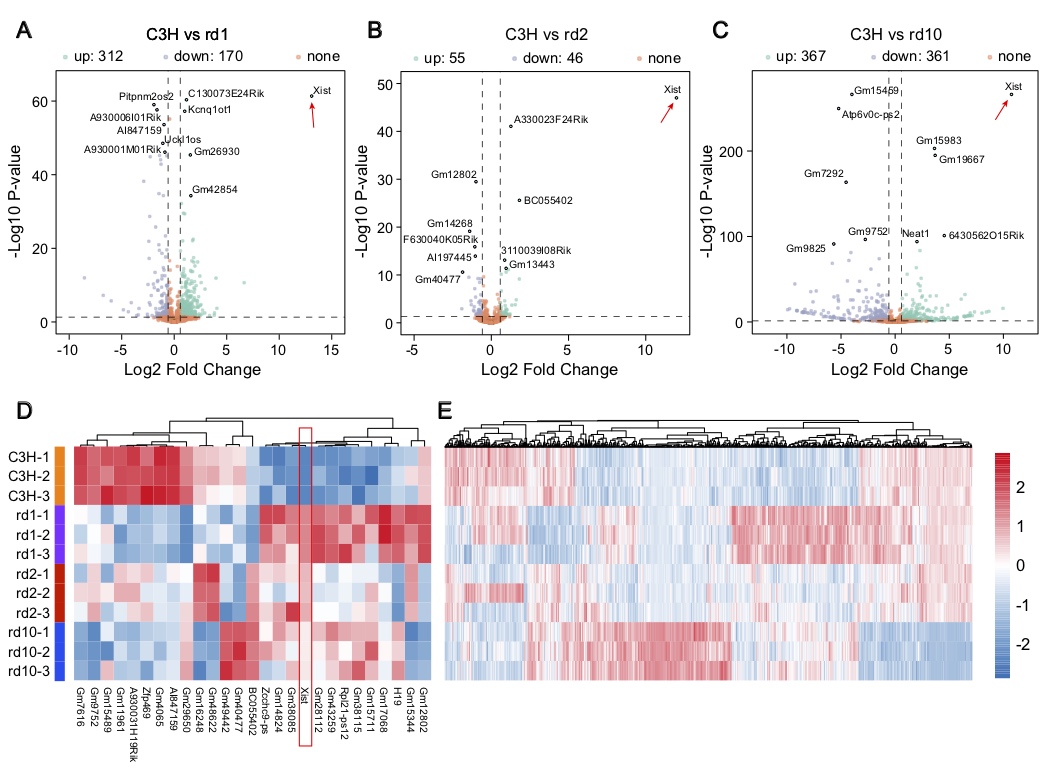

Supplement: Supplementary file 5 [file Image2.JPEG]
