# Supplementary material for: RNA Biological Characteristics at the Peak of Cell Death in Different Hereditary Retinal Degeneration Mutants
Source: Front Genet. 2021 Oct 29;12:728791. doi: 10.3389/fgene.2021.728791 (PMC8586524; doi:10.3389/fgene.2021.728791)
Supplement: Supplementary file 3 [file Table1.DOCX]

Table S1. Statistical summary of the transcriptomic sequencing data.

| **Sample** | **Raw reads** | **Raw bases** | **Clean reads** | **Clean bases** | **Q20(%)** | **Q30(%)** | **NN(%)** | **GC content(%)** |
| --- | --- | --- | --- | --- | --- | --- | --- | --- |
| C3H-1 | 38202605 | 11.46G | 36404232 | 10.92G | 97.2 | 92.54 | 0 | 48.12 |
| C3H-2 | 45703624 | 13.71G | 43581775 | 13.07G | 97.34 | 92.86 | 0 | 48.4 |
| C3H-3 | 38024446 | 11.41G | 35274568 | 10.58G | 97.92 | 94.1 | 0 | 48.93 |
| rd1-1 | 48991734 | 14.70G | 46978977 | 14.09G | 97.5 | 93.08 | 0 | 46.99 |
| rd1-2 | 38992305 | 11.70G | 36900485 | 11.07G | 97.4 | 92.92 | 0 | 47.37 |
| rd1-3 | 43057358 | 12.92G | 41014575 | 12.30G | 97.43 | 92.96 | 0 | 46.26 |
| rd2-1 | 45952323 | 13.79G | 43525633 | 13.06G | 97.91 | 94.08 | 0 | 47.9 |
| rd2-2 | 48355428 | 14.51G | 45429422 | 13.63G | 97.39 | 92.94 | 0 | 49.07 |
| rd2-3 | 47219804 | 14.17G | 45426138 | 13.63G | 97.29 | 92.69 | 0 | 46.75 |
| rd10-1 | 49960975 | 14.99G | 47609379 | 14.28G | 97.43 | 93 | 0 | 47.66 |
| rd10-2 | 37449058 | 11.23G | 35547637 | 10.66G | 97.89 | 94.02 | 0 | 48.82 |
| rd10-3 | 40318740 | 12.10G | 38927014 | 11.68G | 97.3 | 92.76 | 0 | 47 |
| Region | 37449058-  49960975 | 11.23G-  14.99G | 35274568-  47609379 | 10.58G-14.28G | 97.20-97.92 | 92.54-94.10 | 0.00-0.00 | 46.26-49.07 |
| Average | 43519033 | 13.06G | 41384986 | 12.42G | 97.49 | 93.15 | 0 | 47.75 |
| Sum | 522228400 | 156.67G | 496619835 | 148.99G |  |  |  |  |
