# Supplementary material for: RNA Biological Characteristics at the Peak of Cell Death in Different Hereditary Retinal Degeneration Mutants
Source: Front Genet. 2021 Oct 29;12:728791. doi: 10.3389/fgene.2021.728791 (PMC8586524; doi:10.3389/fgene.2021.728791)
Supplement: Supplementary file 6 [file Table2.DOCX]

Table S2. Statistical summary of alignment of sequencing data to reference genome.

| **Sample name** | **Total reads** | **Total mapped** | **Uniquely mapped** | **Multiple mapped** | **Discordantly 1 time** |
| --- | --- | --- | --- | --- | --- |
| C3H-1 | 36404232 | 34434763(94.59%) | 32368535(88.91%) | 1238319(3.40%) | 34161(0.09%) |
| C3H-2 | 43581775 | 41202210(94.54%) | 38788641(89.00%) | 1449370(3.33%) | 41154(0.09%) |
| C3H-3 | 35274568 | 33383851(94.64%) | 31368640(88.93%) | 1361968(3.86%) | 29940(0.08%) |
| rd1-1 | 46978977 | 44512580(94.75%) | 42165285(89.75%) | 1274063(2.71%) | 48505(0.1%) |
| rd1-2 | 36900485 | 34782397(94.26%) | 32925359(89.23%) | 1031719(2.80%) | 31208(0.08%) |
| rd1-3 | 41014575 | 38959744(94.99%) | 36982102(90.17%) | 1045718(2.55%) | 40280(0.1%) |
| rd2-1 | 43525633 | 41423344(95.17%) | 39064025(89.75%) | 1515578(3.48%) | 33299(0.08%) |
| rd2-2 | 45429422 | 42580997(93.73%) | 39906682(87.84%) | 1593927(3.51%) | 57141(0.13%) |
| rd2-3 | 45426138 | 43041265(94.75%) | 40596761(89.37%) | 1255027(2.76%) | 80787(0.18%) |
| rd10-1 | 47609379 | 45562175(95.70%) | 43279415(90.91%) | 1506113(3.16%) | 30444(0.06%) |
| rd10-2 | 35547637 | 33983540(95.60%) | 32296450(90.85%) | 1194103(3.36%) | 25522(0.07%) |
| rd10-3 | 38927014 | 37233688(95.65%) | 35432975(91.02%) | 1099215(2.82%) | 40144(0.1%) |
| Region | 35274568-47609379 | 93.73%-95.70% | 87.84%-91.02% | 2.55%-3.86% | 0.06%-0.18% |
| Average | 41384986 | 39258379(94.86%) | 37097905(89.64%) | 1297093(3.13%) | 41048(0.10%) |
