# Supplementary material for: RNA Biological Characteristics at the Peak of Cell Death in Different Hereditary Retinal Degeneration Mutants
Source: Front Genet. 2021 Oct 29;12:728791. doi: 10.3389/fgene.2021.728791 (PMC8586524; doi:10.3389/fgene.2021.728791)
Supplement: Supplementary file 7 [file Table4.DOCX]

Table 4 The sequences of all primers

| Primer | Sequence (5’-3’) |
| --- | --- |
| GAPDH | F: CCTCGTCCCGTAGACAAAATG |
|  | R: TGAGGTCAATGAAGGGGTCGT |
| XIST | F: GTGCTGTGTGAGTGAACCTATGG |
|  | R: TTTATCCAGTCCGCTGTGCTC |
| H2K2 | F: CTCCTCTATCCACCGACTCCAA |
|  | R: CACAGGGAACATCAGATACTTGAC |
| KLF6 | F: GGTGACAAGGGTAATGGCGA |
|  | R: CGGTATGCTTTCGGAAGTGTC |
| MAFF | F: GCTGTGGATCCCTTATCTAGCAA |
|  | R: CAGCTCGGACTTCTGCTTCTG |
| mmu-cicr0000135 | F: AGGAGGAGACGCTAGAGGCTTA |
|  | R: GCGATTGCTTTAATATCTTCCC |
| mmu-cicr0008206 | F: GCCAAGGTCGGAGTCTGTAGAA |
|  | R: CGTTCCTATGAAGCCATCAACA |
